# Digging in real-word electronic database for assessing CDK 4/6 inhibitors adherence in breast cancer patients from Romania

**DOI:** 10.3389/fphar.2024.1345482

**Published:** 2024-02-23

**Authors:** Adina Turcu-Stiolica, Ion Udristoiu, Mihaela-Simona Subtirelu, Victor Gheorman, Madalina Aldea, Elena Adriana Dumitrescu, Simona Ruxandra Volovat, Dragos Mircea Median, Cristian Virgil Lungulescu

**Affiliations:** ^1^ Pharmacoeconomics Department, University of Medicine and Pharmacy of Craiova, Craiova, Romania; ^2^ Psychiatry Department, University of Medicine and Pharmacy of Craiova, Craiova, Romania; ^3^ Doctoral School, Carol Davila University of Medicine and Pharmacy, Bucharest, Romania; ^4^ Department of Medical Oncology, University of Medicine and Pharmacy Grigore T. Popa Iasi, Iasi, Romania; ^5^ Gynecologic Oncology Department, Filantropia Clinical Hospital Bucharest, Bucharest, Romania; ^6^ Oncology Department, University of Medicine and Pharmacy of Craiova, Craiova, Romania

**Keywords:** palbociclib, abemaciclib, ribociclib, CDK 4/6 inhibitors, breast cancer, adherence, proportion of days covered (PDC)

## Abstract

**Introduction:** It is imperative for patients to respect the prescribed treatments to achieve the anticipated clinical outcomes, including the outpatients receiving oral anti-cancer drugs such as selective cyclin-dependent kinase 4/6 inhibitors (CDK 4/6i). With the introduction of three CDK 4/6i drugs in the Romanian pharmaceutical market in 2018, our study aimed to evaluate medication adherence and the influencing factors among patients undergoing treatment with palbociclib, ribociclib, or abemaciclib for advanced or metastatic breast cancer.

**Methods:** Medication adherence was assessed using the Proportion of Days Covered (PDC) method, and Spearman correlation analysis was conducted to explore the relationships between adherence, age, gender, and follow-up duration.

**Results:** The study enrolled 330 breast cancer patients, with an average follow-up period of 14.6 ± 12.5 months for palbociclib, 10.6 ± 7.1 months for ribociclib, and 8.6 ± 6.4 months for abemaciclib-treated patients. A small proportion of patients demonstrated non-adherence: 12.8% for palbociclib, 14.6% for ribociclib, and 14.7% for abemaciclib. Among patients receiving palbociclib, there was no significant correlation between adherence, age (rho = 0.07, *p* = 0.35), or gender (rho = −0.144, *p* = 0.054). However, a significant correlation was found with the duration of follow-up (rho = −0.304, *p* < 0.0001). Similar results were observed for patients receiving ribociclib or abemaciclib. Most patients received combination therapy with letrozole (46%) and exemestane (13%) for palbociclib, letrozole (48%) and fulvestrant (19%) for ribociclib, and fulvestrant (39%) and letrozole (27%) for abemaciclib,

**Discussion:** High adherence rates were observed among patients treated with CDK 4/6i drugs, with no significant differences noted among the three drugs in this class. However, the collected patient data was limited, lacking information on adverse reactions that could potentially lead to treatment discontinuation, as determined by the oncologist’s decision not to prescribe. Consequently, a comprehensive understanding of all factors contributing to the low adherence levels is hindered.

## Introduction

Breast cancer is the second most common cancer in women after skin cancer with a percentage of 15.2% from all new cancer cases and 7.1% from all cancer deaths in 2023 (National Cancer Institute, 2023. https://www.cancer.gov/types/breast). The identification of cyclin-dependent kinases (CDK) and their regulatory mechanisms in cell cycle processes marked a pivotal advancement in cancer therapy. Among these, cyclin-dependent kinase 4 and 6 (CDK4/6) are enzymes crucially involved in cell cycle regulation. They exert significant control over the transition from the G1 (gap 1) phase to the S (synthesis) phase, where DNA replication occurs (Suryadinata et al., 2010). Maintaining a delicate equilibrium between CDK4/6 activation by cyclin D and their inhibition by cyclin-dependent kinase inhibitors (CDKi) is essential for the orderly progression of the cell cycle. Any disruption in this balance can result in uncontrolled cell division, contributing to various diseases, notably cancer (Barnum et al., 2014). In the realm of cancer treatment, CDK 4/6 inhibitors (CDK 4/6i) are employed to target overactive CDK4/6-cyclin D complexes. This is particularly pertinent in cancers like breast cancer, where this pathway often plays a central role in unregulated cell proliferation (Mariotto et al., 2017).

Palbociclib, ribociclib, and abemaciclib stand as prominent examples of CDK 4/6i widely employed in the treatment of specific forms of advanced/metastatic breast cancer (A/mBC) (Roskoski et al., 2019). Although these inhibitors demonstrate efficacy in impeding cancer cell proliferation, they are not devoid of adverse reactions and side effects (Jin et al., 2019). Previous research has indicated that abemaciclib is associated with a lower preference weight in comparison to other CDK4/6i due to adverse events, including diarrhea, abdominal pain, grade 3/4 neutropenia, tromboembolitic disease (Maculaitis et al., 2020), or acute liver injury (Beachler et al., 2021). Additionally, findings from a singular study (Cejuela et al., 2023) underscored diarrhea as a significant adverse reaction experienced by all patients, highlighting its clinical importance (Arbuckle et al., 2000). A meta-analysis regarding the risk of other side effects, such as stomatitis, demonstrated that especially palbociclib, among all CDK4/6i, could increase this risk impacting on patient adherence to the treatment (Long et al., 2021).

The global market for CDK 4/6i drugs is segmented across various categories, including drug types such as palbociclib (@Ibrance), ribociclib (@Kisqali), and abemaciclib (@Verzenio) (Finn et al., 2015). The first CDK4/6 inhibitor drug approved by the FDA was palbociclib in February 2015 (Dhillon et al., 2015; Fin et al., 2016). Subsequent approvals were granted for its utilization in combination with other hormonal therapies, rendering it a pivotal treatment option for specific breast cancer patients. Ribociclib received FDA approval in March 2017 (Hortobagyi et al., 2016). Similar to palbociclib, it was sanctioned for the treatment of hormone receptor-positive (HR+) and human epidermal growth factor receptor 2-negative (HER2-negative) advanced or metastatic breast cancer in conjunction with an aromatase inhibitor. Its scope has been broadened since then, with additional approvals for diverse hormonal therapies (Salmon et al., 2020). Abemaciclib obtained FDA approval in September 2017. It was endorsed as a standalone agent for HR+, HER2-negative advanced or metastatic breast cancer in patients who had previously undergone endocrine therapy (Dickler et al., 2017). According to the submission of its dossier to EMA, abemaciclib was approved in combination with an aromatase inhibitor (AI, as letrozole, anastrozole, or examestan) as initial endocrine-based therapy or in combination with fulvestrant as initial endocrine-based therapy or following endocrine therapy.

These CDK4/6i have substantially enhanced treatment options for patients with HR+ breast cancer by targeting the cell cycle regulation process, which plays a pivotal role in cancer growth (Wells 2020). Typically, they are utilized in combination with endocrine therapies, significantly prolonging progression-free survival (PFS) and overall survival (OS) for numerous patients (Cristofanilli et al., 2016; Finn et al., 2016; Sledge et al., 2017; Slamon et al., 2018). CDK 4/6i are also utilized together with endocrine therapy for male patients diagnosed with HR+/HER2-metastatic breast cancer (Kraus et al., 2022). It is crucial to note that approval dates and availability can vary by country, and new applications and indications for these drugs may have emerged (Bandiera et al., 2023).

In Romania, approximately 12,000 new cases of breast cancer are diagnosed annually, rendering it the second leading cause of cancer-related deaths, following lung cancer (Furtunescu et al., 2021). According to research on the effects of COVID-19 pandemic in Romania on the breast cancer patients, even if the number of patients remained the same, the cancer treatment costs have risen exponentially from 2018 to 2021 (Turcu-Stiolica et al., 2022). Following Health Technology Assessment (HTA), the National Agency for Medicines and Medical Devices (NAMMD) in Romania unconditionally approved the inclusion of palbociclib in the Positive Drug List in November 2017 (Ministry of Health of Romania, 2017). Ribociclib was unconditionally included in the Positive Drug List in August 2022 (Inclusion of ribociclib in Romania, 2022), while abemaciclib was included in April 2022 (Inclusion of abemaciclib in Romania, 2022). All three medications were recommended for the treatment of women with locally advanced or metastatic breast cancer (a/mBC), who are HR+/HER2-, in combination with an AI or fulvestrant, as initial hormonal therapy, or in women who have received prior hormonal therapy. In premenopausal or perimenopausal women, hormonal therapy should be combined with a luteinizing hormone-releasing hormone (LHRH) agonist.

Medication adherence is a hey enabler of best health outcomes and some medication adherence supporting activities were reported in order to guide research and practice on enhancing medication adherence (Kardas et al., 2023). Treatment nonadherence is associated with disease progression and mortality among patients with breast cancer (Chirgwin et al., 2016). The existing research on adherence to CDK4/6i anticancer agents is limited. Consequently, the primary aim of our research was to assess the adherence levels of CDK 4/6i and to explore potential correlations with variables such as age, gender, and the duration of patient follow-up. In addition to this primary objective, our study also sought to investigate potential disparities in medication adherence among the three distinct CDK 4/6i currently available within the pharmaceutical market in Romania. Through this research, we aimed to contribute valuable insights into the patterns of medication adherence and its associations with demographic factors, thereby enhancing our understanding of the real-world usage of these CDK 4/6i in clinical practice from Romania.

## Methods

In the context of our study conducted in Romania, electronic information pertaining to reimbursed medications is exclusively accessible through the database maintained by the Romanian Health Insurance House. Ethical approval for our research endeavor, granted under Ethics Council approval number 175/29.10.2021, allowed us access to anonymized patient data sourced from community pharmacies in Dolj County, Romania, which were reported to the Health Insurance House of Dolj. The study focused on data spanning the past 5 years, from 2018 to 2022, corresponding to the period during which the first CDK 4/6 inhibitor, palbociclib, was approved for entry into the Romanian pharmaceutical market.

Specifically, our study inquired about patient records identified by the ICD-10 code C50, denoting breast cancer, with a subsequent focus on individuals receiving treatment with palbociclib, ribociclib, and abemaciclib. The data obtained for analysis encompassed essential demographic information, namely, age and gender, as well as details concerning prescription refills, including the quantity of medicines dispensed and the dates of prescription release from community pharmacies. Notably, our access to information was limited to these parameters, and we did not have access to additional patient-specific data such as comorbidities or other health covariates. This approach was undertaken within the confines of ethical guidelines and regulations, ensuring the confidentiality and privacy of patient information while enabling us to analyze patterns of CDK 4/6i usage in the studied population. The utilization of this restricted dataset was essential for our investigation into medication adherence and its potential correlations with demographic factors within the Romanian context.

### Study population

All patients with breast cancer (code of disease = 124) who raised their reimbursed prescriptions from a community pharmacy from Dolj County, Romania, in the period 1 January 2018 -31 December 2022. The first patient received the first palbociclib prescription from the community pharmacy in July 2018, and she was a female of 75 years old, whereas for abemaciclib, the first patient was a female of 73 years old, in February 2021. We included all patients who had at least two fills of CDK 4/6i because it is required to compute medication adherence.

CDK 4/6i cycle dates were determined based on the electronic records from the Dolj Health Insurance House for the reimbursed prescriptions written by the oncologist.

### Outcomes

The duration of follow-up was defined as the time in months from the first prescription issuing by the pharmacist in the community pharmacy to the last prescription reimbursed by the Dolj Health Insurance House according to the analyzed period (1 January 2018-31 December 2022). We considered it as the time elapsed from the medication’s starting date to the last treatment’s discontinuation date, which could be death or treatment modification.

There is no universally standardized method for measuring medication adherence. An ISPOR Report authored by Pednekar et al. highlighted the most frequently employed techniques found in the literature, which include self-reported questionnaires, proportion of days covered (PDC), and medication possession ratio (MPR). The PDC is the leading method used to calculate medication adherence using prescription refill data from electronic records at the population level. PDC was defined as the number of days that drugs were available to the patient over a time interval, but it has many formulas (Pednekar et al., 2019). We calculated the adherence using the formula as the report between Σ cycles/months of supply for medication and Σ months between last month of prescription and the first month of prescription. By definition, PDC ranges from 0 to 1. We used the conventional cutoff point of 0.8 to classify the patients into adherent (0.8 ≤ PDC ≤1) and non-adherent (0 ≤ PDC <0.8) patients (Dima et al., 2017).

### Statistical analysis

We conducted descriptive analysis of continuous variables (age, adherence) using means±standard deviations (SD), median and interquartile range (IQR) and range (minimum-maximum) and of categorical variables (gender, categories of age) using frequencies and percentages. Additionally, to demonstrate the potential correlation between medication adherence and age, gender of patients, we calculated the Spearman’s coefficients and visually presented with heatmaps. To evaluate the differences between the characteristics and medication adherence of patients with different treatment, we used Kruskal–Wallis H test for continuous variables and Chi-square test for categorical variables. We visually presented the differences of medication adherence among patients with different treatments using violin graphs. We conducted statistical analysis using GraphPad Prism 10.1 (GraphPad Software Boston, USA), with the statistical significance level set at *p* less than 0.05, two-tailed.

## Results

During the study period from 1 January 2018, to 31 December 2022, a total of 330 patients were prescribed CDK 4/6i. Among these, 180 patients (55%) were administered palbociclib, 82 (25%) received ribociclib, and 68 (20%) were prescribed abemaciclib.

Table 1 summarizes descriptive statistics of patient characteristics, and adherence, for each group of patients, as well as the *p*-value after performing the comparison between them. The median (range) age was 66 (30–90) years for the palbociclib group, 71 (36–92) years for the ribociclib group, and 66 (43–93) years for the abemaciclib group of patients. Most of the patients were more than 60 years old: 70% in palbociclib patients, 79.3% in ribociclib patients and 73.5% in abemaciclib patients. Most of the patients were female, but more male patients were treated with palbociclib (3.3%) than with ribociclib (1.2%) or abemaciclib (1.5%). The follow-up varies significantly between the three groups of patients (*p*-value = 0.004), with higher follow-up for patients treated with palbociclib, because it was earlier introduced on the Romanian pharmaceutical market.

**TABLE 1:** Characteristics of the patients treated with CDK 4/6 inhibitors.

Characteristics	Palbociclibum (n = 180)	Ribociclibum (n = 82)	Abemaciclibum (n = 68)	*p*-value
Age, years mean ± SD	64.88 ± 11.72	68.46 ± 12.37	66.22 ± 11.38	0.068^a^
median (IQR)	66 (57–74)	70.5 (60.75–77)	66 (58.25–73.75)	
range	30–90	36–92	43–93	
Age, frequencies (%)	4 (2.2%)	2 (2.4%)	0	0.621^b^
30–39	16 (8.9%)	6 (7.3%)	6 (8.8%)	
40–49	34 (18.9%)	9 (11.0%)	12 (17.6%)	
50–59	60 (33.3%)	23 (28.0%)	23 (33.8%)	
60–69	47 (26.1%)	27 (32.9%)	18 (26.5%)	
70–79	19 (10.6%)	15 (18.3%)	9 (13.2%)	
80–93				
Gender, female, n (%)	174 (96.7%)	81 (98.8%)	67 (98.5%)	0.498^b^
Adherence mean ± SD	0.925 ± 0.137	0.92 ± 0.15	0.93 ± 0.14	0.368^a^
median (IQR)	1 (0.89–1)	1 (0.88–1)	1 (0.94–1)	
range	0.11–1.00	0.15–1.00	0.43–1.00	
Follow-up, months mean ± SD	14.6 ± 12.5	10.6 ± 7.1	8.6 ± 6.4	0.004**^a^
median (IQR)	10 (5–21.3)	9 (5.25–16.75)	7 (3–13.25)	
range	1–52	1–26	1–21	

^a^ Kruskal–Wallis H test; b, Chi-square test. **, *p*-value <0.01.

**FIGURE 1:**
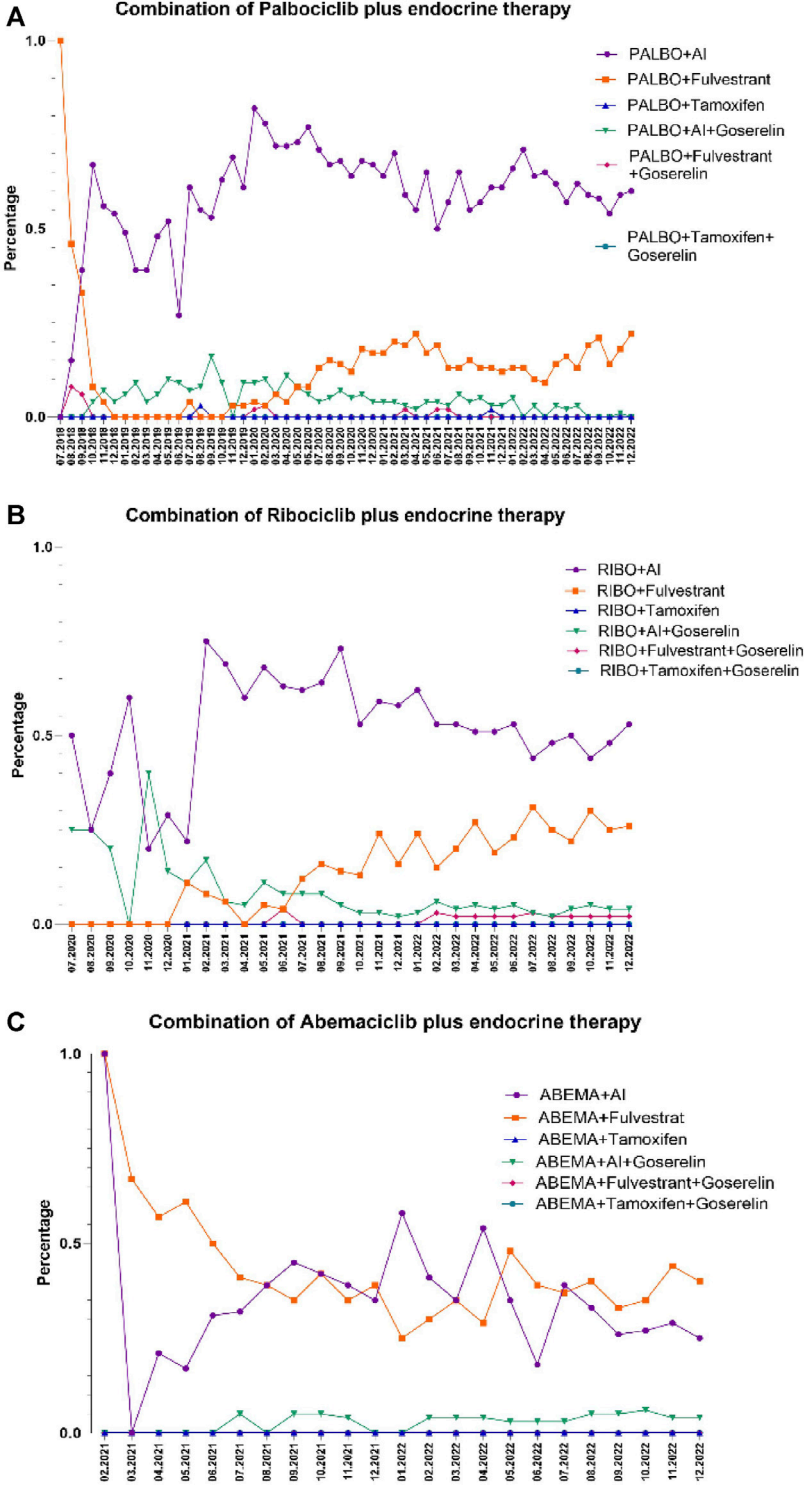
Combination of CDK 4/6i with endocrine therapy by month over the study period. **(A)**. Combination of palbociclib (PALBO) with endocrine therapy. **(B)**. Combination of ribociclib (RIBO) with endocrine therapy. **(C)**. Combination of abemaciclib (ABEMA) with endocrine therapy. AI (aromatase inhibitor). The percentages were computed based on the total number of patients undergoing treatment with both CDK 4/6i and endocrine therapy.

The eligible patients were included in our study with an average follow-up period of 14.6 ± 12.5 months for the patients treated with palbociclib, 10.6 ± 7.1 months for the patients treated with ribociclib, and 8.6 ± 6.4 months for the patients treated with abemaciclib, respectively. CDK 4/6i were generally combined with either letrozole, fulvestrant, exemestane, anastrozole, goserelin or tamoxifen as in Figure 1. No ribociclibum or abemaciclib were combined with tamoxifen in our database. Most of the patients had treatment in combination with letrozole (45.9%) and exemestan (13.4%), in case of palbociclib, letrozole (45.9%) and fulvestrant (19%), in case of ribociclib, and fulvestrant (39.1%) and letrozole (27.4%), in case of abemaciclib, as shown in Table 2. Gosereline was more combined with ribociclibum (5.4%).

**TABLE 2:** Combinations of the CDK 4/6 inhibitors with aromatase inhibitors or/and luteinizing hormone-releasing hormone agonists.

Combination of CDK 4/6i	Palbociclibum (n = 180)	Ribociclibum (n = 82)	Abemaciclibum (n = 68)
Aromatase inhibitors	61.1%	53.7%	33.3%
Letrozole	45.9%	48.4%	27.4%
Anastrozole	1.8%	-	0.7%
Exemestane	13.4%	5.3%	5.38%
Luteinizing hormone-releasing hormone agonist	12.2%	19%	39.1%
Fulvestrant	12.1%	19%	39.1%
Tamoxifen	0.1%	-	-
Aromatase inhibitors + Gosereline	4.4%	5.4%	3.5%
Fulvestrant + Gosereline	0.3%	1.5%	-
Tamoxifen + Gosereline	-	-	-

**FIGURE 2:**
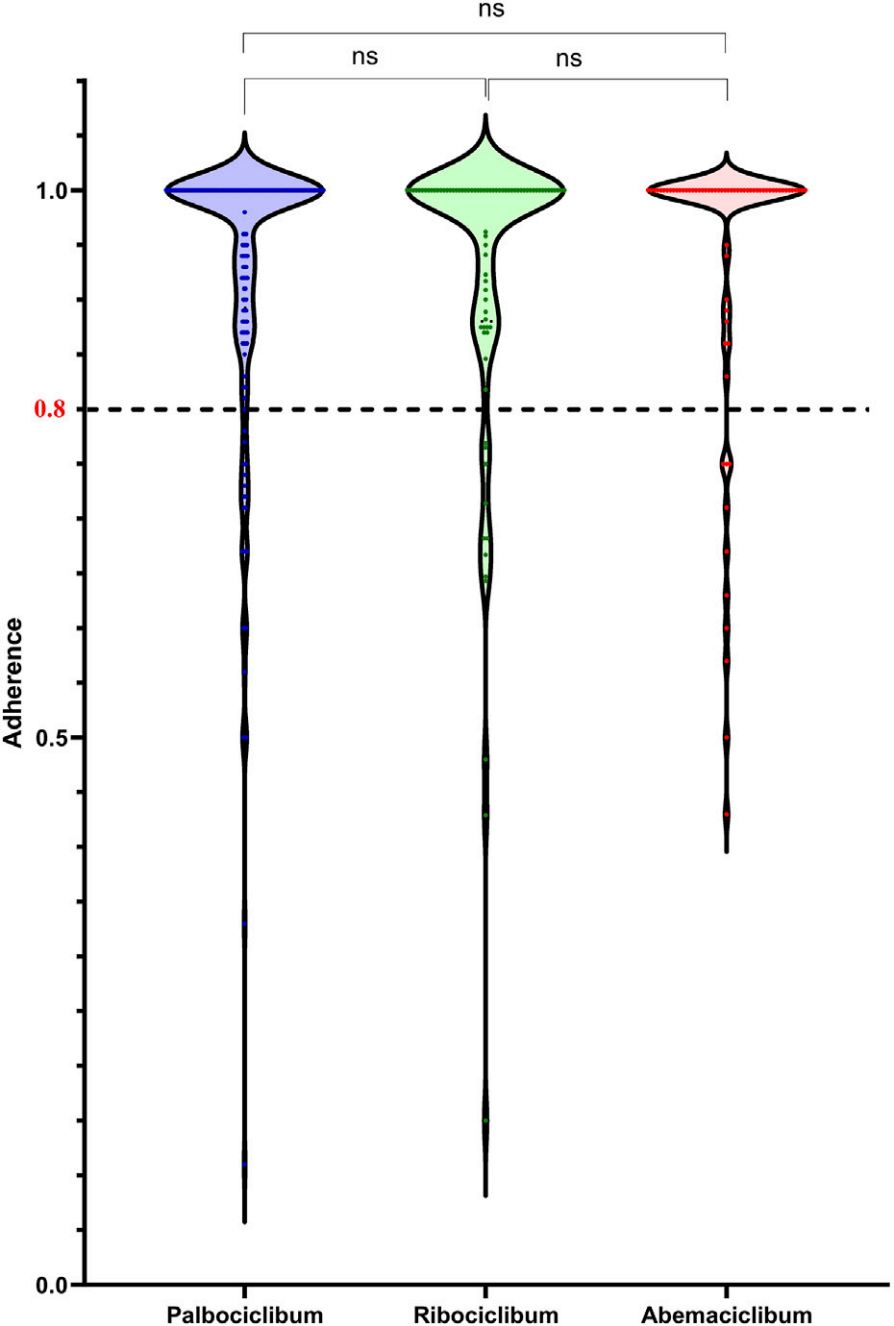
Adherence as proportion of days covered (PDC) in patients treated with either CDK 4/6i, palbociclib, ribociclib, and abemaciclib.

The proportion of non-adherent patients taking CDK 4/6i with PDC <0.8 was 13.6%, splitting into 12.8% for palbociclib, 14.6% for ribociclib, 14.7% for abemaciclib, respectively. For a cut-off equal to 0.85, the proportion of non-adherent patients taking CDK 4/6i was 16.1%, splitting into 16% for palbociclib, 17% for ribociclib, and 16.2% for abemaciclib. For a cut-off equal to 0.90, the proportion of non-adherent patients taking CDK 4/6i was 24.8%, splitting into 25% for palbociclib, 27% for ribociclib, and 22.1% for abemaciclib. No significant difference was obtained for adherence levels among patients treated with the three CDK 4/6i, as shown in Figure 2. We observed the peaks in the CDK 4/6i and the most patients had 100% adherence for all three groups of patients. Better adherence, but not significantly higher, was observed among patients treated with abemaciclib (mean ± SD, 0.93 ± 0.14) than among patients treated with palbociclib (mean ± SD, 0.92 ± 0.14) or ribociclib (mean ± SD, 0.92 ± 0.15). The smallest adherence was observed for a patient treated with palbociclib (0.11), while the smallest adherence observed for a patient treated with ribociclib was 0.15 and the smallest adherence observed for a patient treated with abemaciclib was 0.43.

**FIGURE 3:**
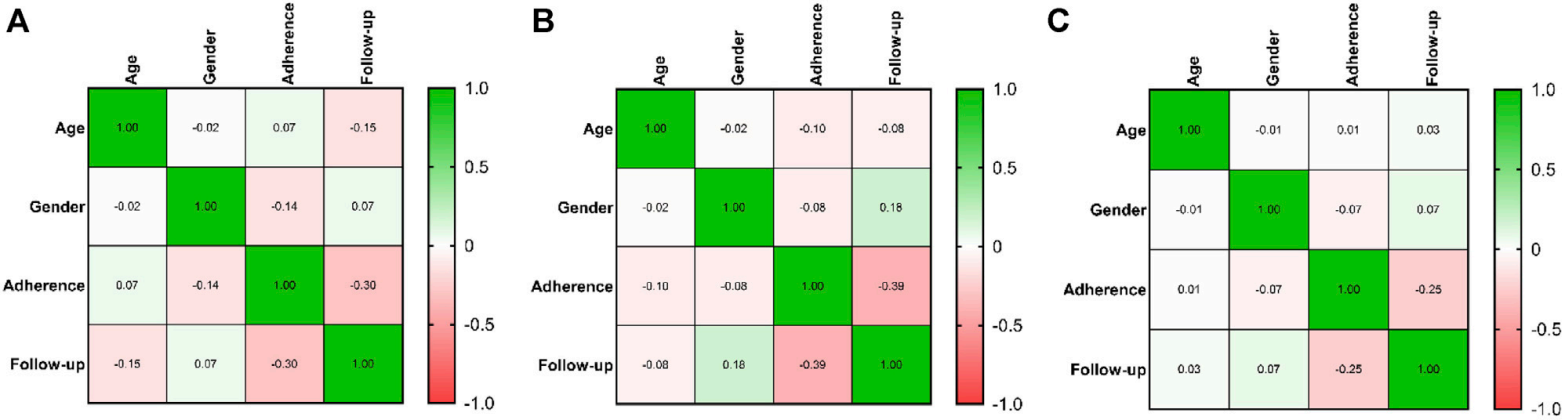
Correlation between adherence of CDK 4/6i treatment, age, gender and follow-up of treatment. The colors from heatmaps correspond to the Spearman coefficient from negative values (light orange color) to positive values (green color). **(A)**. Heatmap of correlations in the case of palbociclib therapy. **(B)**. Heatmap of correlations in the case of ribociclib therapy. **(C)**. Heatmap of correlations in the case of abemaciclib therapy.

As in Figure 3A, in patients treated with palbociclib, there was no significant correlation between the level of adherence, age (rho = 0.07, *p* = 0.35) or gender (rho = −0.144, *p* = 0.054), but a significant correlation was observed with the duration of follow-up (rho = −0.304, *p* < 0.0001). Similarly, in patients receiving ribociclib, no significant correlation was found between adherence levels and age (rho = −0.097, *p* = 0.388) or gender (rho = −0.082, *p* = 0.466), but a significant correlation was identified with the follow-up duration (rho = −0.394, *p* < 0.0001), as is shown in Figure 3B. The same results were obtained for patients treated with abemaciclib, where no significant correlation was found between adherence levels and age (rho = 0.007, *p* = 0.955) or gender (rho = −0.072, *p* = 0.559), but a significant correlation was observed with the duration of follow-up (rho = −0.25, *p* = 0.04), as is shown in Figure 3C.

## Discussion

Maintaining adherence to CDK 4/6i is a mandatory step towards reaching treatment goals for patients with HR+/HER2-a/mBC. We found a proportion of 14% of non-adherent patients taking CDK 4/6i for an 80% adherence cut-off, 16% using an 85% adherence cut-off and 25% using a 90% cut-off, without significant differences between non-adherence for palbociclib, ribociclib and abemaciclib. We obtained an average PDC values of 92.6%, which is comparable with the PDC values of 89.6% obtained by another retrospective study from Canada that included patients receiving either palbociclib or abemaciclib (Marineau et al., 2023). Marineau et al. similar values for mean PDC for palbociclib (90%) and abemaciclib (88.1%), in the same way we obtained for abemaciclib (93%) and palbociclib (92%). Using another method to measure palbociclib adherence, medication possession ratio (MPR), the same results were obtained in a real-world assessment of palbociclib adherence in USA, 88% (Engel-Nitz et al., 2023).

The ribociclib adherence was found to be 92%, similar to the adherence rates measured using patient self-reported questionnaires (87.9%, 91.6%, and 91.6% for EORTC QLQ-C30, QLQ-BR23, and HADS-D, respectively) in RIBANNA trial (Fasching et al., 2022). An ongoing clinical trial LEADER monitored ribociclib adherence by review of patients’ diaries and pill count, without still reported the results (https://clinicaltrials.gov/study/NCT03285412).

Lasala et al. reviewed the studies assessing the association between adherence to oral therapies in cancer patients and clinical outcome and found studies that used different adherence cut-offs that could be associated with different clinical outcomes (Lasala, 2021). None of these studies evaluated CDK 4/6i adherence, but we could compare with studies which included patients with breast cancer under endocrine treatment (tamoxifen, anastrozole, letrozole and exemestane) (Ma et al., 2008; Partridge et al., 2010; Xu et al., 2012; Weaver et al., 2013; Seneviratne et al., 2014; Rodrigues Guedes et al., 2017; Le Saux et al., 2018; Font et al., 2019). Twenty-five percent of non-adherence breast cancer patients were observed in a study that recorded capecitabine adherence by microelectronic monitoring system (MEMS) with a cut-off of 0.80 (Partridge et al., 2010).

The routine of frequent medication intake was proved to be one of the important barriers of adherence to oral anticancer medications among patients with breast cancer (Onwusah et al., 2023). It is important to emphasize that, despite the distinct administration schedules of CDK 4/6 inhibitors (ribociclib and palbociclib are administered once daily for 21 consecutive days followed by 7 days without treatment, while abemaciclib is administered continuously), medication adherence did not differ among the three patient groups.

Seneviratne and Xu showed a statistically significant correlation between medication adherence and OS in breast cancer patients (Xu et al., 2012; Seneviratne et al., 2015). Rodrigues Guedes did not find any correlation (Rodrigues Guedes et al., 2017). Waever et al. did not found significant correlation between adherence and cancer recurrence (Waever et al., 2013). No significant correlation was found between adherence and response according to RECIST (response evaluation criteria in solid tumours) (Le Saux et al., 2018) or relapse-free survival and toxicity (Partridge et al., 2010). Dezentjee et al. demonstrated that tamoxifen adherence was significantly associated with breast cancer event-free time (EFT) for both 80% and 90% adherence cut-offs (Dezentjee et al., 2010).

Few studies were published regarding CDK 4/6i non-adherence negatively effects. Regarding palbociclib adherence, it was measured its impact on pharmacokinetic and pharmacodynamic profiles and proved that catching up on a missed dose at the end of the cycle increases the risk of severe neutropenia in the next cycle (Bandiera et al., 2023).

In our study, we found no significant association between gender and adherence to CDK4/6i, a finding that contrasts with some research indicating gender-specific differences in medication adherence, especially in the context of experiencing adverse effects. For example, a significant difference has been noted in the occurrence of side effects in tamoxifen treatments (Xu et al., 2012). This distinction is important to take into account because the likelihood of side effects is a major factor affecting patients’ compliance with their prescription regimens.

The lack of a gender-based difference in adherence to CDK4/6i in our study is particularly intriguing when juxtaposed with these observations. It prompts further inquiry into the distinctive characteristics of CDK4/6i and their reception and tolerance by different genders and it is important to consider the variety of treatments used for male breast cancer patients.

A study published in Breast in 2022 (Yıldırım et al., 2022) highlights that most male patients were treated with CDK4/6i in combination with fulvestrant or AI rather than tamoxifen. This diverges from the general perception and findings in some interviews (Chalasani, 2023), which suggest that tamoxifen is a more commonly used treatment in male breast cancer patients. This discrepancy in treatment choices is noteworthy because it suggests variability in the clinical management of male breast cancer and potentially different side effect profiles and adherence challenges associated with each treatment.

In our study, among the patients who received palbociclib, 61% patients received a combination with AI and 12.2% a combination with LHRH, in almost the same proportions a US real-world study obtained, 76.1%, palbociclib + AI, and 23.9%, palbociclib + fulvestrant (Engel-Nitz et al., 2022). A study assessing the treatment satisfaction in women receiving palbociclib combination for a/mBC in six countries (USA, Canada, Germany, Netherlands, Argentina, and Denmark) included more patients taking palbociclib plus fulvestrant combination (58.6%), but with a smaller median age than our study–41 years old (Darden et al., 2018). In our study, among the patients who received ribociclib, 53.7% patients received ribociclib + AI and 19% patients received ribociclib + LHRH. Regarding the patients who received abemaciclib, more patients were treated in combination with LHRH (39%) than with AI (33%).

The choice of treatment - whether tamoxifen, fulvestrant, AI + GNRH inhibitors, or CDK4/6i - can have significant implications for adherence. Each medication comes with its own set of potential side effects and impacts on quality of life, which can influence a patient’s willingness and ability to remain adherent. The fact that different treatments are being chosen for male patients in various studies and clinical settings underlines the need for a deeper understanding of how treatment decisions are made and how these decisions affect adherence. This understanding is crucial in developing strategies to improve adherence, especially considering the unique challenges male breast cancer patients may face.

The finding in our study that adherence to CDK4/6i was not significantly associated with age, with most older women showing adherence, is a notable observation in the context of breast cancer treatment.

This outcome aligns interestingly with other publications as the adherence of older women to CDK4/6i in our study is encouraging, especially considering the potential survival benefits highlighted by Petrelli et al. The high adherence rate among older women in our study may reflect the effectiveness of these medications on quality of life, their tolerability, or possibly a good understanding and acceptance of treatment regimens among older patients. This observation is important as it suggests that age alone may not be a significant barrier to adherence in the context of CDK4/6i therapy, emphasizing the need for personalized treatment approaches that account for individual patient profiles rather than solely age-based strategies.

Adherence to CDK 4/6i was significantly associated with the follow-up. This aligns with the findings from other studies (Eliassen et al., 2023) which highlights that adherence and persistence to endocrine treatment are critical for improving event-free and overall survival in non-metastatic breast cancer patients. Therefore, it is plausible that the patients in our study who demonstrated better adherence over extended treatment periods might have experienced improved health outcomes, including longer survival. This potential link between sustained adherence and survival emphasizes the importance of strategies to enhance and maintain adherence in breast cancer treatment. Moreover, on the other side, with extended treatment, patients may begin to see the benefits or stabilization of their condition, reinforcing their trust in the effectiveness of the therapy and motivating them to adhere to the regimen.

Based on these results, different interventions could be developed to enhance CDK4/6i adherence. A mobile health intervention was tested integrating a connected electronic adherence monitoring smartbox and automated texting alerts, resulting a palbociclib adherence of 95.8% ± 7.6% (Sadigh et al., 2023). Baseline, before the intervention, the reported barriers were inconvenience to get prescription filled, forgetfulness, cost, and side effects. Our results regarding the adherence to palbociclib were 92.5% ± 13.7%, but without any interventions and costs could not be among the barriers because the drugs are free, with no out-of-pockets costs. The Romanian National Oncology Program covers these medicines for people diagnosed with cancer, being fully reimbursed by the National Health Insurance House in Romania.

Inherent limitations of real-world analyses using data collected during providing reimbursed drugs include the lack of important information (the stage of the disease), incomplete capture of comorbid conditions, and variations in follow-up/short duration of follow-up.

PDC, as a proxy measure of medication adherence based only on community pharmacy claims data, fails to capture the legitimate reasons for not taking CDK 4/6i drugs and does not measure the patient’s actual medication-taking behavior as self-reported like questionnaires do. Limitations of this study include the unknown reasons for prescribing treatment transient interruptions or cycle start deferrals. Toxicity or adverse effects could be the main reasons. Some adherence barriers were observed in assessing palbociclib adherence: inconvenience to get prescription filled, forgetfulness, cost, and side effects (Sadigh et al., 2023). Despite these limitations from the information extracted from our data sources, our results are the beginning of future research in measuring CDK 4/6i adherence.

Another limitation of our study is associated with the small sample size, as the investigation was conducted exclusively within one of Romania’s counties. Romania lacks patient registries and easily accessible databases. The count of patients in Dolj utilizing CDK4/6i, as reported by the Romanian National Health Insurance House, remained relatively consistent throughout the analyzed years: 8.12% in 2018, 4.72% in 2019, 4.02% in 2020, 4.82% in 2021, and 4.85% in 2022 (calculated as a percentage of the total number of patients using CDK4/6i in Romania). A meta-analysis performing an adjusted indirect comparison among the three CDK 4/6i efficacy and toxicity revealed they are equally effective in either first- or second-line therapy for estrogen receptor-positive advanced breast cancer (Petrelli et al., 2019). Choice of treatment depends on several factors, including patients’ adherence, comorbidities, and disease burden. Despite the limitations of our study, the results do not demonstrate a clear superiority of one of the three CDK 4/6i adherence, further studies are needed to understand the adherence influencing factors and the correlations of clinical outcomes with CDK 4/6i adherence (Huang et al., 2016; Murphy et al., 2012; Rugo et al., 2021).

## Data Availability

The original contributions presented in the study are included in the article/Supplementary material, further inquiries can be directed to the corresponding authors.
